# The complex relationship between physical activity and fatigue with socioeconomic status, and mental health factors in individuals with inflammatory bowel disease

**DOI:** 10.1093/ecco-jcc/jjaf212

**Published:** 2025-11-29

**Authors:** Casandra Dolovich, Sydney Chochinov, Gia Ly, Banke Oketola, Sam Narvey, Sydney Larance, Maitreiy Raman, Sandra C Webber, Charles N Bernstein

**Affiliations:** University of Manitoba IBD Clinical and Research Centre, Winnipeg, R3A1R9, Canada; Department of Internal Medicine, Max Rady College of Medicine, Rady Faculty of Health Sciences, University of Manitoba, Winnipeg, R3A1R9, Canada; University of Manitoba IBD Clinical and Research Centre, Winnipeg, R3A1R9, Canada; Max Rady College of Medicine, Rady Faculty of Health Sciences, University of Manitoba, Winnipeg, R3E0W3, Canada; University of Manitoba IBD Clinical and Research Centre, Winnipeg, R3A1R9, Canada; University of Manitoba IBD Clinical and Research Centre, Winnipeg, R3A1R9, Canada; College of Rehabilitation Sciences, Rady Faculty of Health Sciences, University of Manitoba, Winnipeg, R3E0W3, Canada; University of Manitoba IBD Clinical and Research Centre, Winnipeg, R3A1R9, Canada; Max Rady College of Medicine, Rady Faculty of Health Sciences, University of Manitoba, Winnipeg, R3E0W3, Canada; University of Manitoba IBD Clinical and Research Centre, Winnipeg, R3A1R9, Canada; Department of Medicine, University of Calgary, Calgary, T2N4N1, Canada; College of Rehabilitation Sciences, Rady Faculty of Health Sciences, University of Manitoba, Winnipeg, R3E0W3, Canada; University of Manitoba IBD Clinical and Research Centre, Winnipeg, R3A1R9, Canada; Department of Internal Medicine, Max Rady College of Medicine, Rady Faculty of Health Sciences, University of Manitoba, Winnipeg, R3A1R9, Canada

**Keywords:** fatigue, inflammatory bowel disease, survey, physical activity

## Abstract

**Background:**

We aimed to assess to what extent socioeconomic status (SES) and elevated symptoms of anxiety and depression predict low physical activity (PA) and high fatigue among individuals with inflammatory bowel disease (IBD).

**Methods:**

Participants from the University of Manitoba IBD Research Registry completed a cross-sectional survey pertaining to fatigue, IBD symptoms, PA, and mental health. The International Physical Activity Questionnaire, Modified Fatigue Impact Scale (MFIS), Generalized Anxiety Disorders-7, and Patient Health Questionnaire scales were used. Disease activity was defined by the Inflammatory Bowel Disease Symptom Inventory.

**Results:**

Among those who were fatigued (MFIS > 38) more were <63 years of age (63% vs 49%, *P* < .001), reported education of highschool level or less (34% vs 27%, *P* = .03), had low household income <$50 000 (24% vs. 16%, *P* < .01), were not in a relationship (25% vs 18%, *P* < .001), and were current smokers (16% vs 7%, *P* < .0001). The odds of low SES were greater for those who participated in low PA (OR = 2.75, 95% CI = 1.8-4.3), low PA and were fatigued (OR = 3.05, 95% CI = 1.7-5.3), and low PA excluding fatigue (OR = 2.28, 95% CI = 1.3-3.9). Low SES was not significantly associated with fatigue (*P* = .08), particularly after removing PA observations (OR = 1.00, 95% CI = 0.47-1.97). After adjusting for demographic and clinical factors, the odds of being fatigued were greater among those with elevated anxiety (aOR = 14.4, 95% CI = 9.4-22.4), depression (aOR = 39.6, 95% CI = 24.1-67.2), and active disease (aOR = 6.9, 95% CI = 4.8-9.97). The results did not change when removing low PA from the analysis.

**Conclusions:**

Low SES was a main driver of engaging in low PA (and not high fatigue). Anxiety and/or depression and active disease were drivers of high fatigue (and not low PA).

## 1. Introduction

The incidence and prevalence of inflammatory bowel disease (IBD) worldwide is rising, including in Canada. While the role of physical activity (PA) on the course of chronic diseases has become an increasingly important area of research, little is known about PA in relation to IBD. The uptake of exercise among individuals with IBD is rising;^1^ however, they are still more likely to engage in lower levels of PA and have reduced health-related quality of life (HRQOL).^2^ The benefits of PA on HRQOL have become more widely appreciated in the general population, in individuals with chronic diseases,^3^ and in individuals with IBD.^4^ There is evidence to suggest that PA can potentially reduce the risk of developing IBD and act as a preventative measure in those with Crohn’s disease (CD) and ulcerative colitis (UC).^5^^,^^6^ Though limited, there is some evidence to support the importance of PA in mitigating exacerbating symptoms,^1^^,^^7^ but most reports conclude that more research is required. Common barriers reported among the IBD population regarding engaging in PA include fatigue, abdominal pain, and bowel urgency.^8^^,^^9^ In addition, fatigue is a major symptom of IBD (prevalence of 79%-98%, depending on disease activity state^10^) and mental health can influence both fatigue and PA in the general population.^11^ Anxiety (39%) and depression (23%) are two common psychological symptoms among patients with IBD^12^^,^^13^ that are highly correlated with fatigue,^14^ a more sedentary lifestyle, lower presenteeism at work, and a higher rate of unemployment.^15^

There is a lack of evidence specifically among individuals with IBD that examines the relationship between low socioeconomic status (SES) and low PA. We hypothesize that low SES among individuals with IBD is probably a contributing barrier to PA as it is known that lower PA levels exist amongst the general population of lower SES.^16^^,^^17^ Additionally, compared to the general population, individuals with IBD are more likely to be unemployed,^18^ and hence potentially in a lower SES. The overwhelming rise of exercise participation and benefits resulting from PA both in the general population and among those with chronic diseases is highly motivating to our research team. By identifying barriers and developing strategies to overcome them, we can help improve long-term health and QOL. Further investigation is required to provide evidence on the relationship between SES and PA among individuals with IBD.

The bidirectional relationships between PA and fatigue, between PA and mental health, and between fatigue and mental health can become quite complex and interrelated. In this study we aimed to assess to what extent SES and elevated symptoms of anxiety and depression are predictors of low PA, and high fatigue, both individually and as a combined outcome among those with IBD. A secondary objective was to determine whether the relationship between the predictors of interest and outcome low PA and fatigue was driven primarily by the PA factor or the fatigue factor.

## 2. Methods

Participants from the population-based University of Manitoba IBD Research Registry (*n* = 2740) with updated postal and email addresses were invited to complete a cross-sectional survey with questions pertaining to fatigue, IBD symptoms, PA, and mental health.^19^ All participants from the Registry resided in Manitoba, had a confirmed diagnosis of IBD, and were 18 years or older. Study packages, which contained a letter of introduction, consent form, and a copy of the survey, were sent out at the start of March 2024 by regular mail or email, and responses for this analysis were collected between March and September 2024. The survey study was approved by the University of Manitoba Research Ethics Board.

### 2.1. Demographic factors

Demographic factors included binary age ≥63 years, gender (female, male), low SES (highschool or less and total household income less than $50 000), relationship status (single, not single), disease type (UC, CD), disease duration in years, presence or absence of an ostomy bag, and smoking status. The binary age variable was created to adjust for the greater proportion of older participants with IBD (median age = 63.0 years).

### 2.2. Fatigue

The Modified Fatigue Impact Scale (MFIS) is a modified version of the 40-item Fatigue Impact Scale (FIS) which was developed to assess the effects of fatigue on quality of life in patients with chronic diseases.^20^ The MFIS consists of 21 self-report items, and can be aggregated into three subscales: physical, cognitive, psychosocial, and total MFIS score. All items are scored 0-4 with a minimum total score of 0 and maximum score of 84. A higher score is an indication of greater impact of fatigue on a person’s activities. The total MFIS score was used to define participants as Fatigued (MFIS total score ≥38) vs non-fatigued (MFIS total score <38).^20^

### 2.3. IBD symptoms

The Inflammatory Bowel Disease Symptom Inventory short form (IBDSI-SF) is a modified version of the validated 38-item IBDSI long form (LF) patient-reported outcome measure for IBD symptoms. The IBDSI-SF is a clinical index that has be used to determine symptom severity or symptomatic “active disease.”^21^^,^^22^ The IBDSI-SF consists of 25 self-reported items based on five symptom subscales (bowel symptoms, abdominal discomfort, fatigue, bowel complications and systemic complications).^23^ The items are scored on a scale of 0-4, aside from item 5 (“my stool consistency was generally,” scored 0-2), and item 24 (“do you have a fistula?,” scored 0-1). The total IBDSI score ranges from 0 to 95 where a higher score indicates greater symptom severity. Participants were defined has having “active disease” if their total IBDSI score was greater than 14 for CD or greater than 13 for UC.

### 2.4. Physical activity

The International Physical Activity Questionnaire Short Form (IPAQ-SF) is a validated seven-item instrument designed primarily for assessing physical activity among adults (ages 15-69 years).^24^ Responses to the IPAQ-SF are given in days/week and (hours or minutes)/day during the last 7 days. Individual scores were computed for walking, moderate-intensity activities, and vigorous-intensity activities as well as a total IPAQ score (sum of all three groups of activities). Individual and total IPAQ scores are calculated using an assigned metabolic equivalent (MET) provided by the IPAQ, based on a previous reliability study.^25^ The corresponding MET values are 3.3 for walking, 4.0 for moderate physical activity, and 8.0 for vigorous physical activity. The formula for calculating individual and total IPAQ scores is: (MET value)×(minutes spent participating in PA)×(days spent participating in PA). For example, a participant who engages in 30 min of moderate PA three times a week would be calculated as 4.0×(30 min)×(3 days) = 360 MET/min/week of moderate PA. Continuous scores were converted into categorical: “low,” “moderate,” “high,” and binary “low” vs “moderate/high” physical activity groups. Data processing and cleaning was completed following the IPAQ scoring protocol. Further details regarding the categorization of the IPAQ groups and data processing can be found at https://sites.google.com/view/ipaq/score. Only participants who responded to both the duration and frequency of engaging in PA were included in the study. For the purpose of this study, we assessed PA using the binary (low vs moderate/high) as the outcome variable.

### 2.5. Mental health

Mental health symptoms were assessed using reliable and validated self-report measures for anxiety (Generalized Anxiety Disorders-7 [GAD] ^26^) and depression (Patient Health Questionnaire [PHQ-9]^27^) The GAD-7 is made up of seven items with a total score ranging from 0 to 21. Similarly, the PHQ-9 is made up of nine items with a total score ranging from 0 to 27. Clinically significant mental health symptoms were defined as GAD-7 and PHQ-9 scales ≥10^27^^,^^28^ for defining elevated symptoms of anxiety and depression respectively.

### 2.6. Statistical analysis

Descriptive statistics are presented as percentages stratified by fatigue (MFIS ≤ 38, MFIS > 38), PA (Low, moderate/high), and combined low PA and fatigued (MFIS > 38) [no, yes]. *P*-values were calculated for each comparison of outcome with demographic/clinical variables using Fisher’s Exact test for categorical variables. The relationship between outcomes (1) PA (per IPAQ), (2) fatigue (per MFIS), (3) low PA and high fatigue and predictors (a) elevated anxiety (GAD-7 ≥ 10), (b) elevated depression (PHQ-9 ≥ 10), and (c) active disease (per IBDSI) were assessed using bivariate analysis with Fisher’s Exact test. Unadjusted logistic regression analysis was used to assess which predictors (main factors of interest: low SES, anxiety, depression, active disease) were independently associated with the three outcomes mentioned above as well as a fourth outcome of PA excluding fatigue and, alternatively, fatigue excluding low PA. Finally, multivariate adjusted logistic regression analysis was used to assess the association between each of the outcomes and the predictors of low SES, elevated anxiety, elevated depression, and active disease. Age, gender, disease type, and statistically significant variables from the unadjusted logistic regression were included in the models. Individual models were generated for the predictors anxiety, depression, and active disease due to collinearity.

## 3. Results

Demographic, IBD symptoms, PA, mental health, and fatigue survey questions are provided in Table S1. Among those who were fatigued (MFIS > 38) compared to not fatigued (MFIS ≤ 38) there was a greater proportion of participants <63 years (63% vs 49%, *P* < .001), education of highschool or less (34% vs 27%, *P* = .03), low income <$50 000 (24% vs 16%, *P* = .01), not in a relationship (25% vs 18%, *P* < .001), and current smokers (16% vs 7%, *P* < .0001) (Table 1). The proportion of low SES was more than double for participants who engaged in low PA compared to moderate/high PA (16% vs 7%, *P* < .0001). The proportion of participants engaging in low PA was greater for those who had an ostomy bag versus those who did not have an ostomy bag (12% vs 7%, *P* < .01) and were current smokers versus non-smokers (14% vs 7%, *P* < .01) (Table 1).

**Table 1.** Descriptive statistics by fatigue (MFIS >38) and physical activity (IPAQ).

	Fatigue			Physical activity (IPAQ)			Low PA and MFIS > 38		
	MFIS ≤ 38	MFIS > 38	*P*	Low	Moderate/high	*P*	No	Yes	*P*
	*n* = 775	*n* = 237		*n* = 237	*n* = 775		*n* = 923	*n* = 89	
**Age in years**			**<.001**			.60			.12
** ≥63 years**	381 (49%)	85 (36%)		112 (47%)	354 (46%)		432 (47%)	34 (38%)	
** Missing**	18 (2%)	2 (1%)		7 (3%)	13 (2%)		19 (2%)	1 (1%)	
**Gender**			.06			.08			.18
** Female**	439 (57%)	151 (64%)		150 (63%)	440 (57%)		532 (58%)	58 (65%)	
** Missing**	1 (0%)	0 (0%)		0 (0%)	1 (0%)		1 (0%)	0 (0%)	
**Education**			**.03**			**<.001**			**.02**
** Highschool or less**	209 (27%)	80 (34%)		89 (38%)	200 (26%)		254 (28%)	35 (39%)	
** Missing**	43 (6%)	17 (7%)		43 (6%)	17 (7%)		43 (6%)	17 (7%)	
**Income**			**<.01**			**.0001**			**<.0001**
** Less than $50 000**	122 (16%)	57 (24%)		61 (26%)	118 (15%)		149 (16%)	30 (34%)	
** Missing**	1 (0%)	0 (0%)		1 (0%)	0 (0%)		1 (0%)	0 (0%)	
**Low SES**			.09			**<.0001**			**<.001**
** Yes**	63 (8%)	28 (12%)		39 (16%)	52 (7%)		73 (8%)	18 (20%)	
**Relationship status**			**<.001**			.29			**.01**
** Unclear**	14 (2%)	13 (5%)		6 (3%)	21 (3%)		22 (2%)	5 (6%)	
** Single**	136 (18%)	59 (25%)		54 (23%)	141 (18%)		170 (18%)	25 (28%)	
** Not single**	625 (81%)	165 (70%)		177 (75%)	613 (79%)		731 (79%)	59 (66%)	
**Disease type**			.29			.45			.73
** Ulcerative colitis**	390 (50%)	107 (45%)		111 (47%)	386 (50%)		456 (49%)	41 (46%)	
** Crohn’s disease**	372 (48%)	121 (51%)		121 (51%)	372 (48%)		449 (49%)	44 (49%)	
** Missing**	13 (2%)	9 (4%)		5 (2%)	17 (2%)		18 (2%)	4 (4%)	
**Disease duration (years)**			.21			.13			.33
** Median (IQR)**	29 (18-38)	26 (18-36)		30 (20-39)	27 (18-37)		28 (18-37)	28 (20-40)	
** Missing**	72 (9%)	19 (8%)		25 (11%)	66 (9%)		81 (9%)	10 (11%)	
**Ostomy bag**			.17			**<.01**			.06
** Yes**	57 (7%)	24 (10%)		29 (12%)	52 (7%)		69 (7%)	12 (13%)	
** Missing**	2 (0%)	0 (0%)		0 (0%)	2 (0%)		2 (0%)	0 (0%)	
**Ever smoked**			**<.0001**			**<.01**			**<.001**
** Yes**	53 (7%)	38 (16%)		33 (14%)	58 (7%)		73 (8%)	18 (20%)	
** Missing**	2 (0%)	2 (1%)		1 (0%)	3 (0%)		3 (0%)	1 (1%)	

*P*-values: calculated using Fisher’s exact test for categorical variables. Statistically significant at *P* < .05.

IPAQ, The International Physical Activity Questionnaire; MFIS, The Modified Fatigue Impact Scale; SES, socioeconomic status.

**Figure 1.**
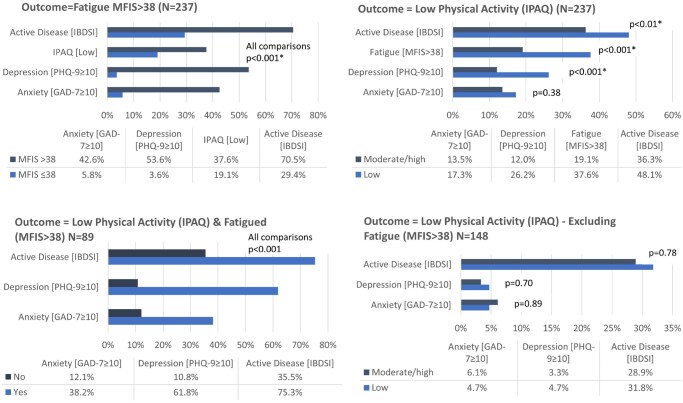
Relationship between physical activity (IPAQ), fatigue (MFIS), and mental health factors anxiety (GAD-7), depression (PHQ-9), and active disease (IBDSI). There is no longer a relationship between low physical activity and clinically significant depression or active disease when we exclude all participants who are extremely tired.

The proportion of elevated symptoms of anxiety (42.6%), depression (53.6%), low PA (37.6%), and active disease (70.5%) were substantially greater for those with high fatigue versus those with low fatigue at 5.8%, 3.6%, 19.1%, and 29.4%, respectively (*P* < .001 for all comparisons; Figure 1). Similarly, participants with low PA were more likely to have depression (26.2% vs 12.0%, *P* < .001), be fatigued (37.6% vs 19.1%, *P* < .001), or have symptomatically active IBD (48.1% vs 36.3%, *P* < .01), compared to patients engaged in moderate/high PA. When observations for participants with fatigue (MFIS > 38) were removed from the analysis, the previous associations were no longer statistically significant. When low PA observations were removed from the analysis, there was no impact on the results above with outcome being fatigue (data not shown).

**Figure 2.**
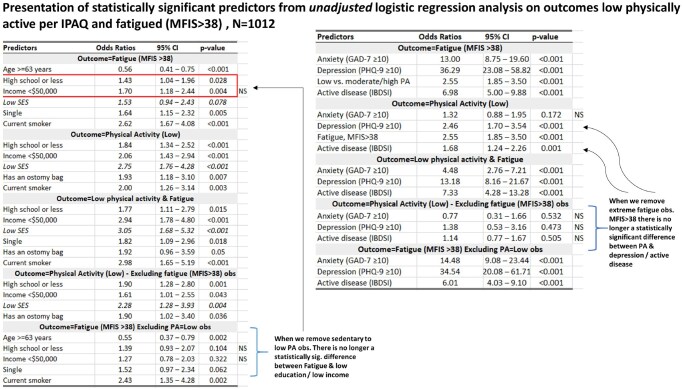
Unadjusted logistic regression analysis of low physical activity (PA), fatigue, and combined low PA and high fatigue with demographic and clinical predictors.

The unadjusted logistic regression analysis results are presented in Figure 2. The odds of low SES were two to three times greater for those who participated in low PA (odds ratio [OR] = 2.75, 95% confidence interval [CI] = 1.8-4.3), or low PA and were fatigued (OR = 3.05, 95% CI = 1.7-5.3), or low PA excluding fatigue (OR = 2.28, 95% CI = 1.3-3.9). Low SES was not statistically significantly associated with the outcome fatigue (*P* = .08). However, both lower education (highschool or less) and lower income <$50 000 (essentially low SES) were significantly greater for participants who were fatigued (OR = 1.43, 95% CI = 1.04-1.96, *P* = .03; and OR = 1.70, 95% CI = 1.18-2.44, *P* = .004, respectively). When low PA observations were removed from the analysis, the predictors highschool or less and income <$50 000 were no longer statistically significant with outcome fatigue.

The odds of elevated mental health symptoms of anxiety (OR = 13.0) and depression (OR = 36.3), as well as symptomatically active disease (OR = 7.0) were greater for those who were fatigued (Figure 2). The results did not change when removing low PA from the analysis. Similarly, the odds of depression and of active disease were 2.5 and 1.7 times greater, respectively, for those who participated in low PA. However, when fatigue observations were removed from the analysis the results were no longer statistically significant. Overall, the odds of elevated anxiety (OR = 4.5, 95% CI = 2.8-7.2), depression (OR = 13.2, 95% CI = 8.2-21.7), and active disease (OR = 7.3, 95% CI = 4.3-13.3) were increased for those with combined high fatigue and low PA (*P* < .001 for all).

Lastly, Table 2 displays the adjusted logistic regression analysis results for all five outcomes predicted by low SES, elevated levels of anxiety and depression, and active symptomatic disease. After adjusting for demographic, clinical, and mental health factors, the odds of low PA and fatigue were greater for low SES (OR = 2.9, 95% CI = 1.5-5.7, *P* < .01), clinically significant anxiety (OR = 4.1 CI = 2.4-6.9, *P* < .001), depression (OR = 12.3, 95% CI = 7.3-21.2, *P* < .001), and active disease (OR = 6.3 CI = 3.6-11.7, *P* < .001). The previous odds ratios were tripled for clinically significant anxiety and depression when the outcome was solely fatigue (OR = 14.4 and 39.6). Excluding low PA observations did not change the results. The association between low SES and low PA did not change when excluding fatigue (MFIS > 38) observations.

**Table 2.** Adjusted logistic regression analysis for outcomes: (A) combined low physical activity (PA) and high fatigue, (B) fatigue only, and (C) low PA only, predicted by low socioeconomic status (SES), clinically significant anxiety and depression, and active symptomatic disease.

A.									
	Outcome = Low physical activity and fatigued (MFIS > 38)								
	MODEL 1			MODEL 2			MODEL 3		
Predictors	OR	CI	*P*	OR	CI	*P*	OR	CI	*P*
**Obs. used**	*n* = 882			*n* = 877			*n* = 941		
**(Intercept)**	0.05	0.03-0.09	<.001	0.03	0.01-0.06	<.001	0.03	0.01-0.06	<.001
**Age ≥63 years**	0.71	0.41-1.20	.207	0.91	0.51-1.62	.753	0.65	0.39-1.07	.097
**Female**	1.44	0.86-2.45	.171	1.3	0.76-2.28	.346	1.17	0.71-1.96	.548
**CD**	0.94	0.57-1.55	.804	0.88	0.52-1.49	.642	0.86	0.53-1.41	.558
**Low SES**	2.94	1.46-5.70	**.002**	2.82	1.34-5.72	**.005**	2.02	1.01-3.86	**.039**
**Currently smoke**	2.42	1.20-4.67	**.01**	2.18	1.02-4.47	**.037**	1.83	0.92-3.49	.074
**GAD7 ≥ 10**	4.09	2.42-6.86	**<.001**						
**PHQ9 ≥ 10**				12.29	7.27-21.19	**<.001**			
**Active disease**							6.28	3.57-11.73	**<.001**

B.									
	Outcome = Fatigue (MFIS >38)								
	MODEL 1			MODEL 2			MODEL 3		
Predictors	OR	CI	*P*	OR	CI	*P*	OR	CI	*P*
**Obs used**	*n* = 860			*n* = 856			*n* = 918		
**(Intercept)**	0.11	0.07-0.17	<.001	0.08	0.05-0.13	<.001	0.11	0.07-0.16	<.001
**Age ≥63 years**	0.79	0.53-1.17	.241	0.97	0.62-1.52	.877	0.57	0.40-0.82	**.002**
**Female**	1.31	0.88-1.94	.182	1.24	0.80-1.95	.335	1.05	0.74-1.51	.769
**CD**	1.22	0.83-1.80	.302	1.08	0.70-1.66	.738	1.05	0.74-1.49	.772
**Low SES**	1.41	0.74-2.61	.284	0.85	0.40-1.75	.675	0.95	0.53-1.68	.862
**Single**	1.52	0.96-2.40	.073	1.58	0.92-2.65	.09	1.47	0.96-2.23	.07
**currently smoke**	2.13	1.16-3.84	**.013**	2.83	1.47-5.31	**.001**	1.72	1.00-2.93	**.048**
**GAD7 ≥ 10**	14.37	9.37-22.43	**<.001**						
**PHQ9 ≥ 10**				39.57	24.11-67.20	**<.001**			
**Active disease**							6.85	4.78-9.97	**<.001**

	Excluding PA = Low observations								
	MODEL 4			MODEL 5			MODEL 6		
Predictors	OR	CI	*P*	OR	CI	*P*	OR	CI	*P*
Obs used	*n* = 658			*n* = 647			*n* = 705		
**(Intercept)**	0.07	0.04-0.12	<.001	0.07	0.04-0.12	<.001	0.08	0.05-0.14	<.001
**Age ≥63 years**	0.87	0.53-1.43	.594	0.96	0.55-1.66	.877	0.58	0.38-0.89	**.014**
**Female**	1.33	0.82-2.18	.248	1.25	0.73-2.16	.413	1.12	0.73-1.72	.605
**CD**	1.29	0.80-2.09	.293	1.14	0.67-1.94	.621	1.19	0.78-1.81	.426
**Low SES**	1.27	0.50-2.96	.588	0.48	0.14-1.45	.219	0.76	0.31-1.70	.525
**Single**	1.73	0.97-3.06	.06	1.45	0.74-2.75	.272	1.67	0.99-2.76	**.049**
**Currently smoke**	1.75	0.76-3.81	.172	1.81	0.73-4.22	.183	1.45	0.72-2.83	.287
**GAD7 ≥ 10**	16.76	10.09-28.42	**<.001**						
**PHQ9 ≥ 10**				34.5	19.34-64.14	**<.001**			
**Active disease**							6.1	3.97-9.56	**<.001**

C.									
	Physical activity (low vs moderate/high)								
	MODEL 1			MODEL 2			MODEL 3		
Predictors	OR	CI	*P*	OR	CI	*P*	OR	CI	*P*
Obs used	*n* = 875			*n* = 952			*n* = 939		
**(Intercept)**	0.19	0.13-0.27	<.001	0.18	0.12-0.25	<.001	0.16	0.11-0.24	<.001
**Age ≥63 years**	1.17	0.83-1.63	.37	1.05	0.76-1.45	.751	1.12	0.81-1.54	.505
**Female**	1.32	0.95-1.84	.105	1.35	0.98-1.87	.07	1.33	0.97-1.85	.082
**CD**	0.96	0.69-1.32	.785	0.98	0.71-1.34	.876	0.97	0.71-1.34	.869
**Low SES**	2.52	1.52-4.15	**<.001**	2.24	1.37-3.64	**.001**	2.24	1.38-3.61	**.001**
**Ostomy bag**	1.56	0.90-2.65	.105	1.48	0.85-2.53	.155	1.59	0.93-2.66	.082
**Currently smoke**	1.58	0.94-2.63	.08	1.66	0.99-2.73	**.049**	1.6	0.96-2.63	.068
**PHQ9 ≥ 10**	2.11	1.41-3.13	**<.001**						
**Active disease**				1.45	1.06-2.00	**.021**			
**fatigue (MFIS >38)**							2.23	1.58-3.14	**<.001**

	Excluding fatigue (MFIS > 38) observations								
	MODEL 4			MODEL 5					
Predictors	OR	CI	*P*	OR	CI	*P*	OR	CI	*P*
Obs used	*n* = 677			*n* = 725					
**(Intercept)**	0.16	0.10-0.24	<.001	0.15	0.09-0.22	<.001			
**Age ≥63 years**	1.25	0.84-1.85	.268	1.2	0.82-1.77	.351			
**Female**	1.34	0.90-2.00	.149	1.43	0.97-2.12	.073			
**CD**	1.03	0.69-1.52	.902	1.04	0.71-1.53	.839			
**Low SES**	2.03	1.08-3.70	**.023**	1.98	1.07-3.59	**.026**			
**Ostomy bag**	1.56	0.79-2.97	.181	1.61	0.81-3.07	.158			
**currently smoke**	1.57	0.77-3.04	.195	1.76	0.88-3.38	.097			
**PHQ9 ≥ 10**	0.93	0.30-2.39	.896						
**Active disease**				1.03	0.68-1.54	.886			

Table A represents three separate adjusted logistic regression models with outcome = Low physical activity and fatigue adjusted by MODEL 1: age, gender, disease type, low SES, smoking status and anxiety GAD7 ≥ 10.

MODEL 2: age, gender, disease type, low SES, smoking status and depression (PHQ9 ≥ 10).

MODEL 3: age, gender, disease type, low SES, smoking status, and active disease.

CI, 95% confidence interval; OR, odds ratio

Table B represents six separate adjusted logistic regression models with outcome = Fatigue (MFIS > 38).

MODEL 1: age, gender, disease type, low SES, relationship status, smoking status, and anxiety GAD7 ≥ 10.

MODEL 2: age, gender, disease type, low SES, relationship status, smoking status, and depression (PHQ9 ≥ 10).

MODEL 3: age, gender, disease type, low SES, relationship status, smoking status, and active disease.

MODELS 4–6 repeat MODELS 1–3 excluding PA = Low observations.

CI, 95% confidence interval; OR, odds ratio.

Table C represents five separate adjusted logistic regression models with outcome = Physical activity (low vs moderate/high).

MODEL 1: age, gender, disease type, low SES, presence of an ostomy bag, smoking status, and depression (PHQ9 ≥ 10).

MODEL 2: age, gender, disease type, low SES, presence of an ostomy bag, smoking status, and active disease.

MODEL 3: age, gender, disease type, low SES, presence of an ostomy bag, smoking status, and fatigue (MFIS >38).

MODEL 4: same as MODEL 1 excluding fatigue (MFIS > 38) observations.

MODEL 5: same as MODEL 2 excluding fatigue (MFIS > 38) observations.

CI, 95% confidence interval; OR, odds ratio.

## 4. Discussion

This study highlights two interesting insights: low SES was a main driver of engaging in low PA (and not high fatigue) in a Manitoban IBD population. The second key finding was that anxiety and/or depression (up to 16.8 and 39.6 times greater, respectively) and active disease (up to 6.9 times greater) were drivers of high fatigue (and not low PA). There was an association between lower education (highschool or less), and lower household income <$50 000 and outcome of fatigue, but once low PA observations were removed these associations were no longer statistically significant. For the outcome low PA, the association between low PA and low SES was not affected by the removal of fatigue observations. On the other hand, low PA was also associated with the predictors depression and active symptomatic disease until fatigue observations were removed. This indicates, first, that the association between the predictor SES and outcome low PA and fatigue was mainly driven towards the outcome PA. Second, the association between the predictors clinically significant anxiety/depression, and active disease and outcome low PA and fatigue primarily impacted the fatigue outcome. It is important to note that with the cross-sectional nature of our study we cannot know which came first, elevated anxiety/depression, active disease, or fatigue.

Secondary findings were that individuals with IBD who were <63 years of age, not in a relationship, and current smokers were more likely to be fatigued compared to not fatigued. In addition, participants who had an ostomy bag and were current smokers were more likely to engage in low PA compared to moderate/high PA.

Extreme fatigue in individuals with IBD has been shown to be strongly associated with symptoms of anxiety, depression (measured using GAD-7 and PHQ-9) as well as with active IBD.^29^ However, the role that mental health and fatigue play in predicting PA remains less clear in this population. Elsewhere, a recent cross-sectional study aimed to investigate the relationship between PA levels, fatigue, and other HRQOL in Chinese individuals with IBD.^30^ The authors found that PA was significantly and negatively associated with fatigue (*r* = −.224, *P* < .001), positively correlated with quality of life (*r* = .171, *P* < .01), and sleep disorders were positively associated with both anxiety (*r* = .340, *P* < .01) and depression (*r* = .354, *P* < .001). Another observational cross-sectional study aimed to evaluate the prevalence of anxiety and depression in patients with inactive IBD and to identify factors associated with them.^14^ They found from multivariate analysis that anxiety was significantly associated with fatigue (OR 4.39, 95% CI: 1.22-15.79, *P* = .02) and lower HRQOL (OR 2.46, 95% CI: 1.70-3.91, *P* < .001). Depression was also associated with fatigue (OR 9.70, 95% CI: 1.67-56.27, *P* = .01).

There is little known about the relationship between low SES and PA among individuals with IBD. However, one prospective study aimed to evaluate the association between exercise and subsequent active disease among individuals with IBD in remission.^31^ The results indicated that lower education (highschool or lower) was greater among individuals with IBD in remission who engaged in lower PA compared to higher PA for both CD (8% vs 5%, *P* < .01) and UC (10% vs 2%, *P* < .01). In addition, lower education (highschool or lower) was greater among CD individuals with disease activity at 6 months (9%) compared to CD individuals in remission (6%) (*P* < .01). This suggests that lower education could be linked to reduced PA and potentially higher disease activity in individuals with CD.

### 4.1. General barriers to PA

Fatigue, abdominal pain, joint pain, increased bowel urgency, and fear of worsening symptoms are common barriers to PA among individuals with IBD.^32^ Some barriers can be more easily addressed; for example, in the case of bowel urgency, recommending exercise with a known toilet location such as a home-based exercise or virtual personal training is an attainable solution. For individuals with abdominal or joint pain, after speaking with a practitioner and/or physical therapist, one might recommend low- to moderate-intensity PA such as walking, low-intensity yoga, or resistance strength training.

### 4.2. Low SES

Compared to the general population, individuals with IBD are known to be more likely to be unemployed.^18^ For obvious reasons, low SES poses a challenge in sustaining exercise aspects of PA such as attending gyms, purchasing equipment, personal training access, etc, not to mention the impact low SES has on PA in general. However, the daily advances in technology allow much to be readily available online and free programs using artificial intelligence. For individuals with IBD in a lower SES, it is important to advocate for realistic PA such as getting out for a daily walk. Other inexpensive options may be swimming (if a home pool is available with nearby bathroom), yoga, biking, and simple resistance training with bands (<$10) and a pair of 10 lb dumbbells for $30-$40 CAD. We have previously shown that individuals of low SES have worse outcomes with regard to their course of IBD in terms of hospitalization, critical care admissions, and even death.^33^

With regard to fatigue impacted by anxiety and depression, it is recommended to prioritize sleep and engage in regular PA, adopt a balanced diet, and consider mindfulness practices or therapy.^34^ While fatigue is a barrier to PA, being physically active can reduce fatigue. A systematic review and meta-analysis revealed that PA interventions largely reduced fatigue among adults with chronic conditions.^35^ To summarize, among individuals with IBD, elevated levels of anxiety and depression lead to exhaustion, and exhaustion is negatively correlated with PA. Further, since PA has been shown to reduce fatigue, stress, and anxiety among individuals with IBD,^36^ prioritizing regular exercise as a lifestyle modification could lead to less fatigue, depression, and less anxiety and to a higher quality of life.^37^

As discussed above with regard to PA, exercise programs specifically tailored for individuals with IBD should be made affordable and easily accessible whether that be in-person or online access. More research is needed to fully understand the relationship between SES, PA, and IBD. This is important for developing effective strategies to promote PA among individuals with IBD, regardless of their SES background.

Our study is one of the largest to date to assess the role of PA among individuals with IBD. Our findings are an important contribution in the development of effective strategies to promote PA among individuals with IBD, regardless of their SES background. We used validated tools regarding PA (IPAQ), fatigue (MFIS), and mental health (GAD-7, PHQ-9) that have been repeatedly used in other populations. Our self-reported survey failed to include some important information such as ongoing arthritis or other immune diseases that may contribute to fatigue. A recurring limitation with cross-sectional studies, however, is that it cannot be determined which came first in the relationship between IBD and low PA/fatigue. Over half of the total sample did not respond, so we are not entirely certain how representative our sample is of all individuals with IBD, although the registry is a population-based cohort and not drawn from a referral clinic. Furthermore, our respondents were on average older (mean of 62 years of age), but with the aging of the IBD population in Canada we have a large sample of an important segment of the IBD population at large and it is very important to understand PA and fatigue in this cohort.

## 5. Conclusions

Increased levels of anxiety and depression among the IBD population are highly correlated with high fatigue (and not low PA). By contrast, low SES is predictive of lower levels of PA (and not high fatigue). Improving PA levels among individuals with IBD could potentially reduce clinical anxiety/and or depression and enhance energy levels.

## Supplementary Material

jjaf212_Supplementary_Data

## Data Availability

The datasets presented in this article are not readily available. All available data are presented herein.
