# Supplementary material for: The complex relationship between physical activity and fatigue with socioeconomic status, and mental health factors in individuals with inflammatory bowel disease
Source: J Crohns Colitis. 2025 Nov 29;19(12):jjaf212. doi: 10.1093/ecco-jcc/jjaf212 (PMC12729914; doi:10.1093/ecco-jcc/jjaf212)
Supplement: jjaf212_Supplementary_Data [file jjaf212_supplementary_data.docx]

**Supplementary Material**

**Table S1.** Survey Questions

**Demographic Questions (D)**

**D1. Date of Birth (Month & Year – mm/yyyy) :** _______________________________________

**D2. What was your sex assigned at birth:**

- Male
- Female
- Intersex
- Other, please specify ________________________________
- Prefer not to answer

**D3. My gender identity is (select all that apply):**

- Man
- Woman
- Non-binary/ Transgender/ Gender Diverse
- Other, please specify _________________________________
- Prefer not to answer

**D4. What is the highest level of education that you have completed?**

- Less than high school
- High school degree or equivalent
- College certificate/ Diploma
- University Undergraduate Degree
- Masters or Doctoral degree
- Other certificate or degree

**D5. What is your current employment status?** **(Select all that apply)**

- Full time work (>30 hours/week)
- Part time work (< 30 hours/week)
- Homemaker
- Parental leave
- Full time student
- Part time student
- Unemployed and looking for work
- Unemployed and NOT looking for work
- Retired
- On disability insurance

**D6. Please select which of the following categories represents your TOTAL household income over the last 12 months:**

- Less than $20,000
- $20,000 to $49,999
- $50,000 to $74,999
- $75,000 to $99,999
- $100,000 or more
- Prefer not to answer

**D7. What is your current relationship status? (select all that apply):**

- Single, never married
- Casually dating
- Exclusively dating
- Common law
- Engaged
- Married
- Separated
- Divorced
- Widowed
- My relationship status is not listed. My relationship status is: ______________________
- Prefer not to answer

**IBD Diagnosis Questions (IBD)**

**IBD1. What type of Inflammatory Bowel Disease (IBD) do you have?**

- Crohn’s disease
- Ulcerative colitis
- Ulcerative proctitis
- Indeterminate colitis or type unclassified

**IBD2. Year of IBD diagnosis:** __________________________________________________

**IBD3. Have you ever had surgery to create an ileoanal pouch/ J pouch?**

- Yes
- No

**IBD4. Have you ever had surgery to remove a portion of your colon or small bowel?**

- Yes
- No

**IBD5. Do you have an ostomy bag? (colostomy, ileostomy, etc.)**

- Yes
- No

**IBD Symptom Questions (S)**

**S1. Currently, is your IBD active or in remission?**

- My disease is active (I am experiencing symptoms)
- My disease is in remission (I am not in a flare)

**S2.** If in question S1 you answered that you are currently in **remission**: **When did you last experience a flare? (Month & Year):** _____________________________

**S3. IBDSI – inflammatory bowel disease symptom inventory (short form)**

The following questions ask more specific details about any inflammatory bowel disease-related symptoms you might have. Please rate how frequent or severe the symptoms that you experienced were in the following areas **over the past week.**

1. **My health was (check one):**

- Very good
- Good
- Slightly below par
- Poor
- Very poor
- Terrible

1. **I had abdominal pain (check one):**

- None
- Mild
- Moderate
- Prolonged/ Severe

1. **On average, the number of bowel movement I had each day was: (select one)**

- Less than one a day
- 1
- 2
- 3
- 4
- 5
- 6
- 7
- 8
- 9
- 10 or more

1. **The number of loose/ liquid bowel movements or diarrhea I had most days was:**

- None
- Some, but less than one a day
- 1
- 2
- 3
- 4
- 5
- 6
- 7
- 8
- 9
- 10 or more

1. **My stool consistency was generally (select one):**

- Formed
- Loose
- Liquid

1. **I noticed blood in my stool (select one):**

- None
- Trace amounts
- Obvious bleeding

1. **I had loss of appetite:**

- None
- Mild
- Moderate
- Prolonged/ Severe

1. **I had nausea:**

- None
- Mild
- Moderate
- Prolonged/ Severe

1. **I had vomiting (select one):**

- None
- Mild
- Moderate
- Prolonged/ Severe

1. **I had tenderness in my abdomen when touched (select one):**

- None
- Minimal
- Moderate
- Severe
- So severe that I pull away when touched

1. **Has your doctor told you that you have a mass or lump in your abdomen?**

- No
- Yes

1. **In the past week, I had a lump in my abdomen:**

- No
- Maybe, hard to tell
- Definitely
- Definitely, and it is tender when touched

1. **How much difficulty have you had with the following during the** **past week**?

|  | None | A little | Moderate | Quite a lot | Severe |
| --- | --- | --- | --- | --- | --- |
| Feeling fatigued or tired and worn out |  |  |  |  |  |
| Excessive bowel gas (farting) |  |  |  |  |  |
| Losing control of bowel movements |  |  |  |  |  |
| Abdominal bloating |  |  |  |  |  |
| Waking because of urge to have bowel movements |  |  |  |  |  |
| Waking because of abdominal pain |  |  |  |  |  |
| Urgency of bowel movements |  |  |  |  |  |
| Finding it hard to get things done |  |  |  |  |  |
| Difficulty releasing gas |  |  |  |  |  |
| Soiling underwear |  |  |  |  |  |
| Abdominal cramps |  |  |  |  |  |

During the **past week,** have you had any of the following complications of IBD? (Terms your doctor might use are in italics.)

1. **Joint pain (*arthralgia/arthritis)*:**

- None
- Mild
- Moderate
- Severe

1. **Do you have a fistula? (a fistula is a path from the bowel to other organs, or from the bowel to the outside skin)**

- Yes
- No

1. **In the past week, my fistula was sore, swollen, or draining:**

- N/A, I do not have a fistula
- No
- A little
- Moderately
- Quite a lot
- Severely

**Medication Questions (M)**

**M1. Do you currently take any medications for your Inflammatory Bowel Disease?**

- Yes
- No

**M2. If you answered YES to M1, which of the following medications are you currently on for your IBD? (Select all that apply):**

- 5-ASA / mesalamine (includes sulfasalazine, asacol, mezavant, salofalk, pentasa)
- Azathioprine / immuran / 6-mercaptopurine / purinthol
- Prednisone
- Budesonide or entocort
- Enema (5-ASA like salofalk or pentasa or steroid enema like entocort or cotenema)
- Suppositories (5-ASA like salofalk or pentasa)
- Methotrexate
- Infliximab (remicade, inflextra or renflexis)
- Adalimumab (Humira)
- Vedolizumab (Entyvio)
- Ustekinumab (Stelara)
- Tofacitinib (Xeljanz)
- Metronidazole (Flagyl)
- Ciprofloxacin
- Imodium
- Lomotil
- Cholestyramine (Questran or Olestyr or Colestid)
- Tylenol with codeine (like Tylenol 3)
- Narcotics (like Percocet, hydromorphone, morphine, dilaudid, fentanyl patch, Demerol)
- Other (please specify): __________________________________________

**M3. Do you use any alternative health products for your Inflammatory Bowel Disease, not prescribed by your gastroenterologist?**

- Yes
- No

**M4. If you answered YES to M3, which kinds of alternative health products do you use? (select all that apply)**

- St. John’s wort/ millepertuis valierian
- Chamomile
- Ginseng
- Kava Kava/ Kava root/ piper methysticum
- Lavender
- Chasteberry/ chaste tree berries/ vitex agnus-castus
- Black cohosh
- Ginkgo biloba
- New Recover-DA
- Lactobacillus acidophilus (probiotic)
- Vitamins
- Echinacea
- Cannabis
- Other (please specify) ________________________________________________

**Smoking Questions (SM)**

**SM1. Do you currently smoke cigarettes?**

- Yes
- No

**SM2. If you answered YES to question SM1, how many cigarettes do you have on a typical day?** _________ /day

**SM3. If you answered NO to question SM1, have you ever smoked cigarettes?**

- Yes
- No

**SM4.** If in question SM3 you answered YES that you used to smoke cigarettes**: When did you stop smoking? (Month & Year)** ____________________________

**SM5.** If in question SM3 you answered YES that you used to smoke**: How many years did you smoke for?** _________

**International Physical Activity Questionnaire (IPAQ)**

We are interested in finding out about the kinds of physical activities that people do as part of their everyday lives. The questions will ask you about the time you spent being physically active in the **last 7 days.** Please answer each question even if you do not consider yourself to be an active person. Please think about the activities you do at work, as part of your house and yard work, to get from place to place, and in your spare time for recreation, exercise or sport.

Think about all the **Vigorous** activities that you did in the **last 7 days.** Vigorous physical activities refer to activities that take hard physical effort and make you breathe much harder than normal. Think *only* about those physical activities that you did for **at least 10 minutes** at a time.

1. **During the last 7 days, on how many days did you do Vigorous physical activities like heavy lifting, digging, aerobics, or fast bicycling?**

**#**________ Days/week **If NO vigorous physical activities **SKIP TO QUESTION (3)**

1. **How much time did you usually spend doing Vigorous physical activities on one of those days?**

**#**________ Hours/day  **Or #**________Minutes/day

- Don’t know/ Not sure

Think about all the **Moderate** activities that you did in the **last 7 days.** Moderate activities refer to activities that take moderate physical effort and make you breathe somewhat harder than normal. Think only about those physical activities that you did for **at least** **10 minutes** at a time.

1. **During the last 7 days, on how many days did you do moderate** **physical activities like carrying light loads, bicycling at a regular pace, or doubles tennis? Do Not include walking**

**#**________**Days/week** **If NO moderate physical activities **SKIP TO QUESTION (5)**

1. **How much time did you usually spend doing moderate physical activities on one of those days?**

**#**________ Hours/day  **Or #**________ Minutes/day

- Don’t know/ not sure

Think about the time you spent **Walking** in the **last 7 days.** This includes at work and at home, walking to travel from place to place, and any other walking that you have done solely for recreation, sport, exercise, or leisure.

1. **During the last 7 days, on how many days did you Walk for at least 10 minutes at a time?**

**#**________ Days/week **If NO walking in the past 7 days **SKIP TO QUESTION (7)**

1. **How much time did you usually spend Walking** **on those days?**

**#**________ Hours/day  **Or #**________Minutes/day

- Don’t know/ not sure

The last question is about the time you spent **Sitting** on weekdays during the **last 7 days.** Include time spent at work, at home, while doing course work and during leisure time. This may include time spent sitting at a desk, visiting friends, reading, or sitting or lying down to watch television.

1. **During the last 7 days, how much time did you spend Sitting** **on a week-day?**

**#**________ Hours/day  **Or #**________ Minutes/day

- Don’t know/ not sure

**The following two definitions are important to note when answering questions below on Physical Activity (questions PA1 – PA7), and Exercise (questions E1 – E8)**

1. **PHYSICAL ACTIVITY:** refers to movement of the body, and includes activities such as taking the stairs, walking for leisure, doing yard work, household chores, etc.
2. **EXERCISE:** a branch of physical activity that includes scheduled/structured movement, such as aerobic training, resistance training, walking for exercise, organized sport, etc.

**Physical Activity Questions (PA)**

FIRST, we are curious about how your IBD impacts your **PHYSICAL ACTIVITY.** Please select the answers below that best apply to you.

**PA1. What types of physical activity do you participate in most frequently? (Select all that apply):**

- Household cleaning (e.g. sweeping, mopping, vacuuming, etc.)
- Laundry/ tidying up your home
- Taking out the trash/ recycling
- Grocery shopping (in-store)
- Carrying groceries
- Gardening (e.g. planting, weeding, watering, etc.)
- Shoveling snow
- Brushing snow off vehicle
- Raking leaves
- Mowing lawn
- Pool care
- Playing with children/ taking care of children
- Taking the stairs (e.g. at work, when shopping, at home, etc.)
- Walking for leisure (e.g. walking a dog, walking around at work, walking to bus stop, etc.)
- **N/A** – I do not participate in any physical activity

If you participate in any other forms of physical activity not mentioned above, please list below: ____________________________________________________________________________________________________________________________________________________________

**REMINDER:** Physical Activity (PA) refers to movement of the body, and can include activities such as taking the stairs, walking for leisure, doing yard work, household chores, etc.

**PA2. Are there any types of physical activity that you AVOID, specifically due to your IBD? (Select all that apply):**

- Household cleaning (e.g. sweeping, mopping, vacuuming, etc.)
- Laundry/ tidying up your home
- Taking out the trash/ recycling
- Grocery shopping (in-store)
- Carrying groceries
- Gardening (e.g. planting, weeding, watering, etc.)
- Shoveling snow
- Brushing snow off vehicle
- Raking leaves
- Mowing lawn
- Pool care
- Playing with children/ taking care of children
- Taking the stairs (e.g. at work, when shopping, at home, etc.)
- Walking for leisure (e.g. walking a dog, walking around at work, walking to bus stop, etc.)
- I avoid **ALL** physical activity
- **N/A** – I do not avoid any physical activity due to my IBD

If you avoid any other types of physical activity specifically due to your IBD, please list below: ____________________________________________________________________________________________________________________________________________________________

**REMINDER:** Physical Activity (PA) refers to movement of the body, and can include activities such as taking the stairs, walking for leisure, doing yard work, household chores, etc.

**PA3. In general, my IBD (select all that apply):**

- **NEVER** impacts my physical activity level
- Causes me to **reduce** my physical activity level, but only when I HAVE ibd symptoms
- Causes me to **reduce** my physical activity level, but only when I have NO ibd symptoms
- Causes me to **reduce** my physical activity level, whether I have ibd symptoms or not
- Causes me to **avoid** physical activity, but only when I HAVE ibd symptoms
- Causes me to **avoid** physical activity, but only when I have NO ibd symptoms
- Causes me to **avoid** physical activity all together (whether I have ibd symptoms or not)
- Causes me to **increase** my physical activity level, but only when I HAVE ibd symptoms
- Causes me to **increase** my physical activity level, but only when I have NO ibd symptoms
- Causes me to **increase** my physical activity level, whether I have ibd symptoms or not

**PA4.** If in question PA3, you answered that you reduce/avoid physical activity due to your IBD: **What factors influence your decision to reduce/avoid physical activity? (Select all that apply)**

- The impact of symptoms (e.g. abdominal pain, bloating, joint pain, fatigue, etc.)
- Concerns of having a flare
- Concerns of bathroom access
- Concerns of passing gas when around other people
- Concerns it may worsen symptoms
- Having an ostomy
- Not knowing what physical activity I can safely perform
- Having to cancel plans without warning
- Concerns with body image
- Time constraints

If due to other reasons, please elaborate below:

____________________________________________________________________________________________________________________________________________________________

**REMINDER:** Physical Activity (PA) refers to movement of the body, and can include activities such as taking the stairs, walking for leisure, doing yard work, household chores, etc.

**PA5**. If in question PA4, you answered that your SYMPTOMS lead you to reduce/avoid physical activity: **Which symptoms in particular cause you to reduce/avoid physical activity? (Select all that apply)**

- Abdominal pain
- Bloating
- Gas
- Bowel urgency
- Diarrhea
- Constipation
- Joint pain/ discomfort
- Nausea/vomiting
- Muscle weakness
- Fatigue
- Skin changes (rashes/ sores)
- Mental health (worry/anxiety/depression)
- Other (please specify) ________________________________________________

**PA6.** If in question PA3, you answered that you **increase** your physical activity due to your IBD: **What factors influence your decision to increase your physical activity?**

________________________________________________________________________________________________________________________________________________________________________________________________________________________________________________________________________________________________________________________

**PA7. Select the statement that best applies regarding the amount of physical activity you do with your IBD:**

- I currently perform less physical activity than I would like
- I currently perform more physical activity than I would like
- I am happy with the amount of physical activity I currently perform

**Exercise Questions (E)**

Now, we are curious about how your IBD impact your ability to **EXERCISE.** Please choose the answers below that best apply to you.

**REMINDER:** Exercise (E) refers to a branch of physical activity that includes scheduled/structured movement, such as aerobic training, resistance training, walking for exercise, organized sport, etc.

**E1. What types of exercise do you participate in most frequently? (Select all that apply):**

- Walking for exercise (e.g. walking for a specified time and/or at a specific pace)
- Running/ jogging
- Cycling outdoors/ spin classes
- Elliptical/ stair master/ rowing machine, etc.
- Body weight exercises (e.g. sit-ups, pull-ups, lunges, squats, plank, etc.)
- Weight lifting (e.g. bicep curls, weighted squats, cable machine exercises, etc.)
- Group sports (e.g. hockey, baseball, volleyball, soccer, etc.)
- Swimming
- Tennis/ squash/ racquet ball/ pickleball, etc.
- Cross country skiing/ snow-shoeing
- Downhill skiing/ snowboarding
- Skating
- Karate/ jiu-jitsu/ judo, etc.
- Kickboxing/ boxing
- Golf
- Yoga
- Pilates
- Dance/ Zumba/ barre/ aerobic classes
- Stretching
- **N/A** – I do not participate in any exercise

If you participate in any other forms exercise not mentioned above, please list below: ____________________________________________________________________________________________________________________________________________________________

**REMINDER:** Exercise (E) refers to a branch of physical activity that includes scheduled/structured movement, such as aerobic training, resistance training, walking for exercise, organized sport, etc.

**E2. Are there any types of exercise that you AVOID, specifically due to your IBD? (Select all that apply):**

- Walking for exercise (e.g. walking for a specified time and/or at a specific pace)
- Running/ jogging
- Cycling outdoors/ spin classes
- Elliptical/ stair master/ rowing machine, etc.
- Body weight exercises (e.g. sit-ups, pull-ups, lunges, squats, plank, etc.)
- Weight lifting (e.g. bicep curls, weighted squats, cable machine exercises, etc.)
- Group sports (e.g. hockey, baseball, volleyball, soccer, etc.)
- Swimming
- Tennis/ squash/ racquet ball/ pickleball, etc.
- Cross country skiing/ snow-shoeing
- Downhill skiing/ snowboarding
- Skating
- Karate/ jiu-jitsu/ judo, etc.
- Kickboxing/ boxing
- Golf
- Yoga
- Pilates
- Dance/ Zumba/ barre/ aerobic classes
- Stretching
- I avoid **ALL** exercise
- **N/A –** I do not avoid any exercise due to my IBD

If you avoid any other types of exercise specifically due to your IBD, please list below: ____________________________________________________________________________________________________________________________________________________________

**E3. In general, my IBD (select all that apply):**

- **NEVER** impacts my ability to exercise
- Causes me to **reduce** the amount of exercise I do, but only when I HAVE ibd symptoms
- Causes me to **reduce** the amount of exercise I do, but only when I have NO ibd symptoms
- Causes me to **reduce** the amount of exercise I do, whether I have ibd symptoms or not
- Causes me to **avoid** exercise, but only when I HAVE ibd symptoms
- Causes me to **avoid** exercise, but only when I have NO ibd symptoms
- Causes me to **avoid** exercise all together (whether I have symptoms or not)
- Causes me to **increase** the amount of exercise I do, but only when I HAVE ibd symptoms
- Causes me to **increase** the amount of exercise I do, but only when I have NO ibd symptoms
- Causes me to **increase** the amount of exercise I do, whether I have ibd symptoms or not

**E4.** If in question E3, you answered that you **reduce/avoid** exercise due to your IBD: **What factors influence your decision to reduce/avoid exercise? (Select all that apply)**

- The impact of symptoms (e.g. abdominal pain, bloating, joint pain, fatigue, etc.)
- Concerns of having a flare
- Concerns of bathroom access
- Concerns of passing gas when around other people
- Concerns it may worsen symptoms
- Having an ostomy
- Not knowing what exercise I can safely perform
- Having to cancel plans without warning
- Concerns with body image
- Time constraints
- Financial concerns (e.g. related to joining a gym, buying sports equipment, etc.)

If due to other reasons, please elaborate below:

____________________________________________________________________________________________________________________________________________________________

**REMINDER:** Exercise (E) refers to a branch of physical activity that includes scheduled/structured movement, such as aerobic training, resistance training, walking for exercise, organized sport, etc.

**E5**. If in question E4, you answered that your SYMPTOMS lead you to reduce/avoid exercise: **Which symptoms in particular cause you to reduce/avoid exercise? (Select all that apply)**

- Abdominal pain
- Bloating
- Gas
- Bowel urgency
- Diarrhea
- Constipation
- Joint pain/ discomfort
- Nausea/vomiting
- Muscle weakness
- Fatigue
- Skin changes (rashes/ sores)
- Mental health (worry/anxiety/depression)
- Other (please specify) ________________________________________________

**E6.** If in question E3, you answered that you **increase** your exercise due to your IBD: **What factors influence your decision to increase your exercise?**

________________________________________________________________________________________________________________________________________________________________________________________________________________________________________________________________________________________________________________________

**E7. Select the statement that best applies regarding the amount you exercise you do with IBD (select one):**

- I currently perform less exercise than I would like
- I currently perform more exercise than I would like
- I am happy with the amount of exercise I am currently perform

**REMINDER:** Exercise (E) refers to a branch of physical activity that includes scheduled/structured movement, such as aerobic training, resistance training, walking for exercise, organized sport, etc.

**E8. Have you ever sought out exercise advice from a personal trainer and/or physiotherapist?**

- Yes, but before my IBD diagnosis
- Yes, I have sought out exercise advice since receiving my IBD diagnosis
- No

**Mental Health Questions (MH)**

**MH1. GAD-7**

Over the **last 2 weeks,** how often have you been bothered by the following problems?

|  | Not at all | Several days | More than half the days | Nearly every day |
| --- | --- | --- | --- | --- |
| Feeling anxious, nervous, or on edge |  |  |  |  |
| Not being able to stop or control worrying |  |  |  |  |
| Worrying too much about different things |  |  |  |  |
| Trouble relaxing |  |  |  |  |
| Being so restless that it’s hard to sit still |  |  |  |  |
| Becoming easily annoyed or irritable |  |  |  |  |
| Feeling afraid as if something awful might happen |  |  |  |  |

**If you checked off any problems in the table above, how difficult have these made it for you to do your work, take care of things at home, or get along with other people? (select one)**

- not difficult at all
- somewhat difficult
- very difficult
- extremely difficult

**MH2. PHQ-9**

Over the last 2 weeks, how often have you been bothered by any of the following problems?

|  | Not at all | Several days | More than half the days | Nearly every day |
| --- | --- | --- | --- | --- |
| Little interest or pleasure in doing things |  |  |  |  |
| Feeling down, depressed, or hopeless |  |  |  |  |
| Trouble falling or staying asleep, or sleeping too much |  |  |  |  |
| Feeling tired or having little energy |  |  |  |  |
| Poor appetite or overeating |  |  |  |  |
| Feeling bad about yourself – or that you are a failure or have let yourself or your family down |  |  |  |  |
| Trouble concentrating on things, such as reading the newspaper or watching television |  |  |  |  |
| Moving or speaking so slowly that other people could have noticed? Or the opposite – being so fidgety or restless that you have been moving around a lot more than usual |  |  |  |  |
| Thoughts that you would be better off dead or of hurting yourself in some way |  |  |  |  |

**If you checked off any problems in the table above, how difficult have these made it for you to do your work, take care of things at home, or get along with other people? (select one)**

- not difficult at all
- somewhat difficult
- very difficult
- extremely difficult

**Fatigue Questions (F)**

**F1. Modified Fatigue Impact Scale**

The following is a list of statements that describe the effects of fatigue. Please read each statement, and choose the choose the answer that best indicates how often fatigue has affected you in this way over the past **4 weeks.**

|  | Never | Rarely | Sometimes | Often | Almost Always |
| --- | --- | --- | --- | --- | --- |
| 1. I have been less alert |  |  |  |  |  |
| 1. I have had difficulty paying attention for long periods of time |  |  |  |  |  |
| 1. I have been unable to think clearly |  |  |  |  |  |
| 1. I have been clumsy and uncoordinated |  |  |  |  |  |
| 1. I have been forgetful |  |  |  |  |  |
| 1. I have had to pace myself in my physical activities |  |  |  |  |  |
| 1. I have been less motivated to do anything that requires physical effort |  |  |  |  |  |
| 1. I have been less motivated to participate in social activities |  |  |  |  |  |
| 1. I have been limited in my ability to do things away from home |  |  |  |  |  |
| 1. I have trouble maintaining physical effort for long periods |  |  |  |  |  |
| 1. I have had difficulty making decisions |  |  |  |  |  |
| 1. I have been less motivated to do anything that requires thinking |  |  |  |  |  |
| 1. My muscles have felt weak |  |  |  |  |  |
| 1. I have felt physically uncomfortable |  |  |  |  |  |
| 1. I have had trouble finishing tasks that require thinking |  |  |  |  |  |
| 1. I have had difficulty organizing my thoughts when doing things at home or at work |  |  |  |  |  |
| 1. I have been less able to complete tasks that require physical effort |  |  |  |  |  |
| 1. My thinking has been slowed down |  |  |  |  |  |
| 1. I have had trouble concentrating |  |  |  |  |  |
| 1. I have limited my physical activities |  |  |  |  |  |
| 1. I have needed to rest more often or for longer periods of time |  |  |  |  |  |
